# Efficacy evaluation of chimeric antigen receptor-modified human peritoneal macrophages in the treatment of gastric cancer

**DOI:** 10.1038/s41416-023-02319-6

**Published:** 2023-06-29

**Authors:** Xuhui Dong, Jiqiang Fan, Wangxu Xie, Xiang Wu, Jia Wei, Zhonglei He, Wenxin Wang, Xueting Wang, Pingping Shen, Yuncheng Bei

**Affiliations:** 1 Department of Oncology, Nanjing Drum Tower Hospital, Affiliated Hospital of Nanjing University Medical School, Nanjing University, 210008 Nanjing, PR China; 2 State Key Laboratory of Pharmaceutical Biotechnology and The Comprehensive Cancer Center, Nanjing Drum Tower Hospital, The Affliated Hospital of Nanjing University Medical School, School of Life Sciences, Nanjing University, 210023 Nanjing, China; 3 Charles Institute of Dermatology, School of Medicine, University College Dublin, Dublin, Ireland; 4 Shenzhen Research Institute of Nanjing University, 518000 Shenzhen, China

**Keywords:** Cancer immunotherapy, Preclinical research, Gastric cancer

## Abstract

**Background:**

Gastric cancer is one of the most common cancers. Peritoneal carcinomatosis (PC) appears to be the most common pattern of recurrence, and more than half of the GC patients eventually die from PC. Novel strategies for the management of patients with PC are urgently needed. Recently, rapid progress has been made in adoptive transfer therapy by using macrophages as the effector cells due to their capabilities of phagocytosis, antigen presentation, and high penetration. Here, we generated a novel macrophage-based therapy and investigated anti-tumoral effects on GC and potential toxicity.

**Methods:**

We developed a novel Chimeric Antigen Receptor-Macrophage (CAR-M) based on genetically modifying human peritoneal macrophages (PMs), expressing a HER2-FcεR1γ-CAR (HF-CAR). We tested HF-CAR macrophages in a variety of GC models in vitro and in vivo.

**Results:**

HF-CAR-PMs specifically targeted HER2-expressed GC, and harboured the FcεR1γ moieties to trigger engulfment. Intraperitoneal administration of HF-CAR-PMs significantly facilitated the HER2-positive tumour regression in PC mouse model and prolonged the overall survival rate. In addition, the combined use of oxaliplatin and HF-CAR-PMs exhibited significantly augment anti-tumour activity and survival benefit.

**Conclusions:**

HF-CAR-PMs could represent an exciting therapeutic option for patients with HER2-positive GC cancer, which should be tested in carefully designed clinical trials.

## Introduction

Despite a global decline in morbidity and mortality over the past 5 decades, GC remains the fifth most common prevalent cancer with more than 1 million new cases diagnosed worldwide each year [1]. Surgical resection remains the best option for patents with early stage of GC. However, in advanced gastric cancer (AGC), intraperitoneal implantation and metastasis of GC is a life-threatening problem with limited effective treatment options and strategies [2]. Up to half of the patients with GC have developed peritoneal carcinomatosis (PC) at the time of initial diagnosis. Effective control is vital for the progression of GC. The currently most indispensable treatment option for AGC is systemic chemotherapy. However, the inability of chemotherapy to target disseminated tumours and the severe toxic side effects on healthy organs make it extremely difficult to benefit AGC patients with PC [3, 4]. Furthermore, GC is characterised by a high degree of heterogeneity that occurs at both inter- and intra-tumour levels, making it prone to develop chemoresistance. Therefore, the development of innovative strategies for the treatment of AGC patients with PC is urgently needed.

Human epidermal growth factor receptor 2 (HER2, also known as ERBB2) proteins are overexpressed in a high proportion of GC cases and affect the maintenance of cancer stem cell subsets [5–7]. Patients with HER2-overexpressing GC benefit from treatment with the anti-HER2 antibodies [1]. Multiple clinical studies of the targeted agent trastuzumab have proved that the application of surface antigen HER2 may serve as a target for adoptive cell transfer (ACT) therapy [8–10]. The ACT of chimeric antigen receptor (CAR) T cell has achieved remarkable success in the treatment of haematological malignancies, and the marketing approval of CAR-T products by the FDA symbolises the new era in cell therapy [11, 12]. ACT with genetically modified T cells has revealed encouraging therapeutic efficacy in haematological tumours, but its application to solid tumours has proven challenging. For example, physical barriers formed by the matrix surrounding tumour cells prevent the entrance of T cells in the tumour environment. In addition, cytokine release syndrome (CRS) induced by multiple cytokines released from CAR-T cells also poses challenges for practical application [13, 14]. These hurdles have spurred the investigation of alternative immune cells as therapeutics.

Macrophages are highly pliable cells with multiple functions, including tumour antigen presentation, tissue homeostasis, clearance of cellular debris, and regulation of inflammatory responses [15, 16]. Macrophages are the central effector and regulator of the human innate immune system. They also play an important role in antitumor immunity due to their phagocytosis, cytotoxicity, pro-inflammatory factor secretion, and antigen presentation to T cells [17, 18]. The ability to infiltrate into both primary tumours and metastases endows macrophage a unique opportunity for cellular therapy [19]. Furthermore, the administration of macrophages appears to be safe, and with no reported high-grade toxicities related to treatments [20]. Modulating macrophages could overcome the limitations of existing CAR-T cells in solid tumours [21]. Attempts to combat cancer using macrophages began decades ago [22–26]. Frustratingly, early clinical trials demonstrated unsatisfactory results of macrophage transplantation therapy [18, 27–30]. This suggests that macrophages require additional modifications optimised to enhance tumour specificity and functional internal signals to direct their activity to eradicate cancer cells. In recent years, researchers have begun paid great interest in developing CAR macrophages (CAR-M) for tumour immunotherapy, offering promising possibilities for the treatment of solid tumours.

The presence of malignant ascites (MA) is a common complication of AGC with PC and usually indicates a poor prognosis [31, 32]. Draining of ascitic fluid is a normal means to relieve discomfort in patients presenting with severe abdominal tightness or shortness of breath [33]. The presence of multiple myeloid cells and lymphocytes within the ascites fluids of patients with peritoneal cancer metastasis make it an important source of ACT cells [34]. Peritoneal macrophages (PMs) comprise a key population of peritoneal resident immune cells, which play crucial roles in recognition and phagocytosis of microbial pathogens, antigen presentation, induction and resolution of inflammation as well as recruitment and activation of other immune cells [35]. Furthermore, PMs isolated from the peritoneal cavity can be utilised in various in vitro assays, including phagocytosis, cytokine production, chemokine production and toxicological studies [36]. Therefore, PMs might be an ideal candidate for the ACT.

Based on our previous work, CAR-modified macrophages showed anti-tumour efficacy by remodelling tumour extracellular matrix and promote T-cell infiltration into the tumour [37]. In this study, we generate genetically modified primary human PMs to target GC cells utilising an anti-HER2 single-chain variable fragment (scFv) derived from HER2-specific monoclonal antibody trastuzumab (Herceptin®) as the antigen-binding domain in CAR structure. The intracellular signal was designed according to the activation mechanism of macrophages to endow PMs with optimised tumour cell phagocytic ability and tumour microenvironment remoulding capacity. The therapeutic efficacy of HER2-GFP-CAR-PMs (HG-CAR-PMs) and HER2-FcεR1γ-CAR-PMs (HF-CAR-PMs) against GC cells were evaluated by constructing the in vitro three-dimensional (3D) GC cell spheres model and the in vivo mouse model of PC. Furthermore, the therapeutic strategy of oxaliplatin combined with HF-CAR-PMs was further evaluated.

## Materials and methods

### Malignant ascites sample collection and study arrangement

Malignant ascites (MA) was collected from three patients (Patient #1, #2 and #3) with highly advanced gastric cancer and underwent palliative paracentesis. Indwelling catheters were used to drain and gain the patient’s ascites. Primary peritoneal macrophages collected from these three patients were all used for construction of CAR macrophages. CAR macrophages derived from Patient #1 was dominantly applied for in vitro investigation, while CAR macrophages derived from Patient #2 and Patient #3 were produced for analysis of in vivo efficacy as monotherapy and combined with oxaliplatin, respectively. However, in vitro cytotoxicity of CAR-macrophage derived from these three patients all has been evaluated. The protocol was approved by the Ethics Committee of Nanjing Drum Tower Hospital.

### Cell culture

Human gastric cancer cell line (MKN45 and HGC-27) and human breast cancer cell line (MDA-MB-231 and MDA-MB-453) were obtained from Shanghai Institutes for Biological Sciences, Chinese Academy of Sciences. MKN45 cells were maintained in RPMI 1640 media. HGC-27, MDA-MB-231, and MDA-MB-453 cells were maintained in DMEM media. All culture media was supplemented with 1% penicillin–streptomycin and 10% foetal bovine serum (Gibco). All cells were cultured at 37 °C with 5% CO_2_.

### Animals

Female BALB/c nude mice (6–8 weeks old) were purchased from the Model Animal Research Center of Nanjing University, Nanjing, China and bred in the animal facilities under specific pathogen-free conditions. The animal studies were approved by the Laboratory Animal Welfare and Ethics Committee of Nanjing University (IACUC-2109011).

### Isolation and identification of peritoneal macrophages

The isolation of peritoneal macrophages from MA has been described [35, 38]. Briefly, MA obtained from GC patients were centrifuged at 1200 rpm for 5 min, and then wash the samples with sterile Hank’s balanced salt solution (HBSS). Add the resuspended cells to the lymphocyte separation solution and centrifuge at 1200 rpm for 30 min. After centrifugation, cells at the monocyte interface were recovered and washed with phosphate buffer solution (PBS). After washing, the obtained cells were centrifuged, resuspended in 1640 medium supplemented with 10% foetal bovine serum, and then incubated in a petri dish at 37 °C with 5% CO_2_ for 1 h. The petri dish was washed twice to remove suspended cells by HBSS. Firmly attached cells were regarded as peritoneal macrophages for subsequent experimental studies. The MA procedure was carried out in accordance with the guidelines verified and approved by the Ethics Committee of Nanjing Drum Tower Hospital. All donors signed an informed consent for the scientific research statement.

### Phagocytosis assays in vitro

Tumour cells were labelled with DiR fluorescent dye before co-culture. Then tumour cells were harvested and resuspended in PBS to a concentration of 1 × 10^6^ cells/mL. Add DiR dye (5 μM) to the cell suspension and incubate at 37 °C for 20 min. After incubation, cells were centrifuged at 1000 rpm for 5 min. Pour off the supernatant, add prewarmed medium to resuspend the cells, and wash slowly for 1–2 times. Then, 1 × 10^5^ control human PMs or CAR-PMs were co-cultured with 1 × 10^5^ DiR-labelled HER2 negative tumour cells or 1 × 10^5^ DiR-labelled HER2^+^ overexpression tumour cells for 6 h. After co-culture, cells were collected, stained with Anti-CD11b-FITC (Anti-CD68-FITC), and analysed by fluorescence-activated cell sorting (FACS). The percentage of cells labelled with DiR in the CD11b^+^ (CD68^+^) population was characterised as the percentage of phagocytosis.

### Cytotoxicity assays in vitro

Tumour cells with luciferase and macrophages were used as target cells (T) and effector cells (E), respectively. The ratio of Effect/Target (E/T) was continuously decreased from 10:1 to 1:10. Bioluminescence was measured using Infinite M200 Pro (Tecan). The percentage of specific lysis was calculated based on the fluorophore enzyme signal relative to the individual tumour using the following equation.

$$\begin{array}{l}\% {{{{{{{\mathrm{Specific}}}}}}}}\;{{{{{{{\mathrm{Lysis}}}}}}}} = \left[ {{{{{{{{\mathrm{Sample}}}}}}}}\;{{{{{{{\mathrm{signal}}}}}}}} - {{{{{{{\mathrm{Tumour}}}}}}}}\;{{{{{{{\mathrm{alone}}}}}}}}\;{{{{{{{\mathrm{signal}}}}}}}}} \right]/\\ \left[ {{{{{{{{\mathrm{Background}}}}}}}}\;{{{{{{{\mathrm{signal}}}}}}}} - {{{{{{{\mathrm{Tumour}}}}}}}}\;{{{{{{{\mathrm{alone}}}}}}}}\;{{{{{{{\mathrm{signal}}}}}}}}} \right] \ast 100.\end{array}$$

### Macrophage targeted killing assay in three-dimensional tumour cell spheroid model

MKN45 cells (1000 cells in 200 μL of complete medium) were added to a 96-well clear round bottom ultra-low attachment microplate (Corning, USA). Three-dimensional (3D) tumour cell spheroids were constructed and cultured, and the experiment could be performed when the cell spheroid diameter has proliferated to about 200–300 μm. The experiment was divided into two groups, the HG-CAR-PM group, and the HF-CAR-PM group. The human peritoneal cells were transfected 1 day before the experiment, and the cells of each group were collected, centrifuged, and washed, then added complete culture medium and resuspended to 5 × 10^4^ cells/mL. Aspirate 100 μL of culture medium from each spheroids culture, add PMs from different groups at an effective target ratio of 5:1 to each culture, and incubate for 16 h, and then add tumour cells in each well. The spheres were carefully aspirated into 1.5 mL EP tubes and washed twice with saline to remove free PMs. The washed tumour cell spheroids were placed in a confocal small petri dish and fluorescence confocal microscopy was performed to observe the penetrating infiltration of PMs in the tumour cell spheres. The images were processed using Image J software 1.37.

### Gene expression analysis

TRIzol reagent (Invitrogen, USA) was used to extract total RNA from macrophages or tumour samples. Total RNA was reverse transcribed into cDNA using the 5 × All-In-One RT Master Mix kit (abm Cat#G486 Code Q111–02). Real-time PCR was performed with a CFX96 real-time PCR detection system (Bio-Rad) using a Q-PCR kit (Vazyme Biotech). Expression levels were calculated by the 2^-ΔΔct^ method compared to the expression of GAPDH. Primers purchased from Genescript (Nanjing, China) are shown in Supplementary Table S1. Macrophages were cultured for 48 h with the normal complete medium was regarded as M0 phenotype; with added LPS (100 ng/mL)/IFN-γ (20 ng/mL) regarded as M1 phenotype; and with added IL-4/IL-13 (10 ng/mL each) regard as M2 phenotype. Phenotypes of polarised macrophages were characterised by quantitative PCR (Q-PCR) and FACS.

### Western blotting

Cells were collected and washed twice with PBS, and proteins were extracted by whole-cell lysis with a kit purchased from Beyotime (Haimen, Jiangsu, China) containing protease and phosphatase inhibitors. Cell debris was removed by centrifugation at 4 °C and protein concentration was determined by Pierce BCA assay. The protein content was electrophoresed on 10% SDS-PAGE gels followed by immunoblotting on polyvinylidene fluoride membrane (American Biosciences). Antibodies used in this study are shown in Supplementary Table S2.

### Flow cytometry analysis

The antibodies are listed in Supplementary Table S1. Flow cytometry was performed and analysed on the ACEA NovoCyte.

### Cytokine release assays

Enzyme-linked immune absorbance assay (ELISA) kits for IL-6, CCL4, TNF-α and IL-1β were purchased from eBioscience (San Diego, CA, USA), and all ELISAs were performed according to the manufacturer’s protocol.

### Mouse peritoneal carcinomatosis model

All animals were purchased from the Model Animal Research Center of Nanjing University (Nanjing, China). The animal studies were approved by the Laboratory Animal Welfare and Ethics Committee of Nanjing University (IACUC-2109011) and carried out at Nanjing University. All animal experiments conformed to the guidelines of the Animal Care and Use Committee of Nanjing University. All efforts to minimise suffering were made. Peritoneal carcinomatosis (PC) was modelled in 6–8-week-old male BALB/c nude mice via injection intraperitoneally with 1 × 10^6^ MKN45 cells. The formation of peritoneal metastasis tumour nodules was assessed by In vivo imaging system.

### In vivo antitumor efficacy

In the MKN45 peritoneal metastasis tumour treatment study, tumour-bearing mice were randomly divided into five groups (*n* = 6) and injected intraperitoneally with 0.2 mL PBS, 1 × 10^7^ HG-CAR-PMs, 1 × 10^6^ HF-CAR-PMs, 1 × 10^7^ HF-CAR-PMs, or 1 × 10^8^ HF-CAR-PMs. Mice were imaged every 7 days using in vivo imaging system. Three weeks after the initiation of treatment, peripheral blood serum was collected for assessment of kidney and liver function. Mice were sacrificed, tumours and organs were excised. To estimate tumour burden, large tumour nodules (tumour bigger than 3 mm in diameter) were weighted and small tumour nodules (tumour smaller than 3 mm in diameter) were counted as described before [39]. One mouse was randomly selected from each group and major organs were collected for histological analysis. Organs were fixed in 10% neutral buffered formalin, embedded in paraffin, sliced, and stained with haematoxylin–eosin (H&E).

### Statistical analysis

GraphPad Prism 9.0 software was used to conduct statistical analyses. All statistics are for two and more independent experiments and are expressed as mean ± standard error (mean ± SEM). When only two groups were compared, statistical analysis of significant differences was performed using the unpaired Student’s *t* test. One-way analysis of variation (ANOVA) followed by Tukey’s multiple comparisons test was used for statistical analysis when the data involved three or more groups for comparison. For all figures, *P* < 0.05, *P* < 0.01, or *P* < 0.001 were considered statistically significant and indicated by *, ** or ***, respectively.

## Results

### Generation of human HER2-specific CAR macrophages

**Fig. 1:**
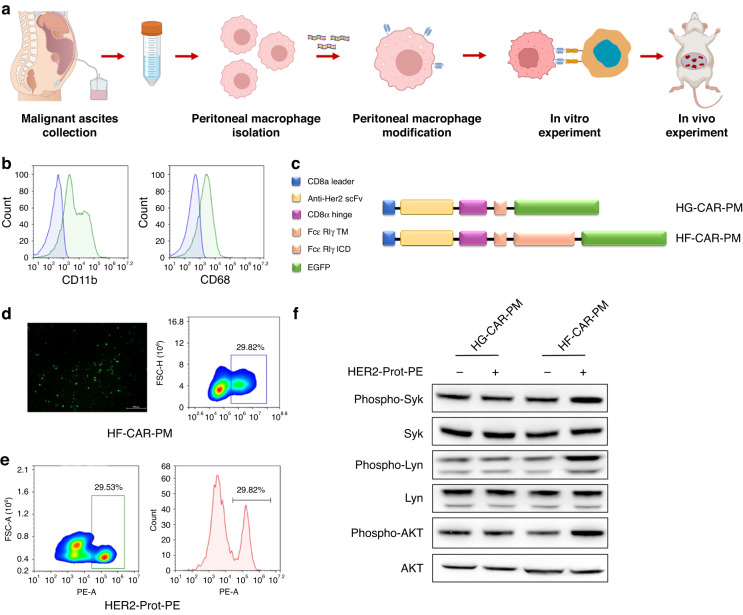
Construction of HER2-specific CAR in primary human peritoneal macrophages. **a** Schematic diagram of experimental design. **b** Flow cytometry analysis of human primary peritoneal macrophages marker CD11b and CD68. **c** Construction of chimeric antigen receptor peritoneal macrophages targeting HER2. **d** Detection of GFP expression in peritoneal macrophages transfected with CAR plasmid by fluorescence microscopy and flow cytometry. **e** Flow cytometry analysis of the binding of PE-conjugated HER2 protein with HER2-specific scFv on the surface of CAR-PMs. **f** Phosphorylation levels of downstream transcription factors Syk, Lyn, AKT of CAR-modified PMs and control cells after HER2 protein stimulation for 48 h.

Since macrophages do not proliferate after injection in vitro or in vivo, patients can only receive limited amounts of macrophages for remodelling and adoptive therapy [40]. It has been previously reported that collected ascites contain a large number of tumour-promoting soluble factors, gastric cancer cells, and immune cells, including large numbers of PMs [41]. Therefore, we speculated that ascites could be used as a source of macrophages for adoptive cell therapy. To this end, we conducted a series of investigation. The main steps of the experimental procedure are shown in Fig. 1a. In our initial effort to generate CAR human macrophages, PMs were obtained from ascites samples of donor 1 (GC Patient #1) with intraperitoneal metastasis and the purity were characterised by macrophage markers CD11b and CD68 by flow cytometry. The results showed that the purity of PMs could reach more than 80% after separation (Fig. 1b). To activate the phagocytic ability of PMs, we generated a codon-optimised HER2-FcεR1γ-CAR (HF-CAR). Briefly, the HF-CAR plasmid was constructed with a CD8a signal peptide, an anti-HER2 single-chain variable fragment (scFv) derived from HER2-specific monoclonal antibody trastuzumab (Herceptin®) [37], a CD8a hinge region, as well as a CD28 transmembrane region (Fig. 1c). In addition, the HF-CAR contains a FcεR1γ intracellular signalling domain and the green fluorescent protein (GFP) reporter gene was used for the detection of CAR expression. The control vector using the same structure as the HER2-specific CAR plasmid but without an intracellular signalling domain named HER2-GFP-CAR (HG-CAR). HF-CAR and the HG-CAR sequences were inserted into the lentiviral vector backbone respectively and subsequently packaged into the lentiviral particles. Transduction efficiency was determined on day 3 with flow cytometry using anti-human HER2 protein and GFP reporter gene. Macrophages are innate immune cells that are not easily transfected by common viral vectors [18]. To overcome the inherent resistance of PMs to lentiviral transfection, we used Vitamin D3 and NATE™ to pretreat and activate macrophages. Notably, the HF-CAR and the HG-CAR transduction efficiency were over 29%, indicating the successful establishment of CAR-macrophage (Fig. 1d). The CAR macrophages derived from Patient #1 were used for subsequent in vitro analysis.

Then, we investigated whether the CAR was activated in HF-CAR-PMs restrictedly after incubation with HER2 antigen. As previous studies reported, PI3K signals play an important role in the internalisation of large targets and promote phagocytosis by macrophages [42]. Then, we evaluated the phosphorylation status of FcεR1γ downstream kinases Syk and Lyn and the activation status of the PI3K/AKT signalling pathway (Fig. 1f). As expected, upon antigen (HER2 recombinant protein) stimulation, HF-CAR-PMs showed a downstream signal activation of FcεR1γ characterised by elevated phosphorylation of Syk and Lyn (Fig. 1f). Moreover, activation-induced cell death, triggered by repeated antigen stimulation, is a major cause of weak persistence of CAR-T cells and restricts its therapeutic efficacy [43–45]. However, our experimental results showed that constant stimulation of HER2 protein does not affect the cell cycle and viability of CAR-PMs (Supplementary Fig. S1A–D). Taken together, these data suggested that HF-CAR was successfully constructed and functionally worked within PMs, while had no effect on macrophage viability.

### FcεR1γ-based CAR activated HF-CAR-PMs polarised toward an anti-tumoral M1-like phenotype and promoted the proliferation of T cells in vitro

**Fig. 2:**
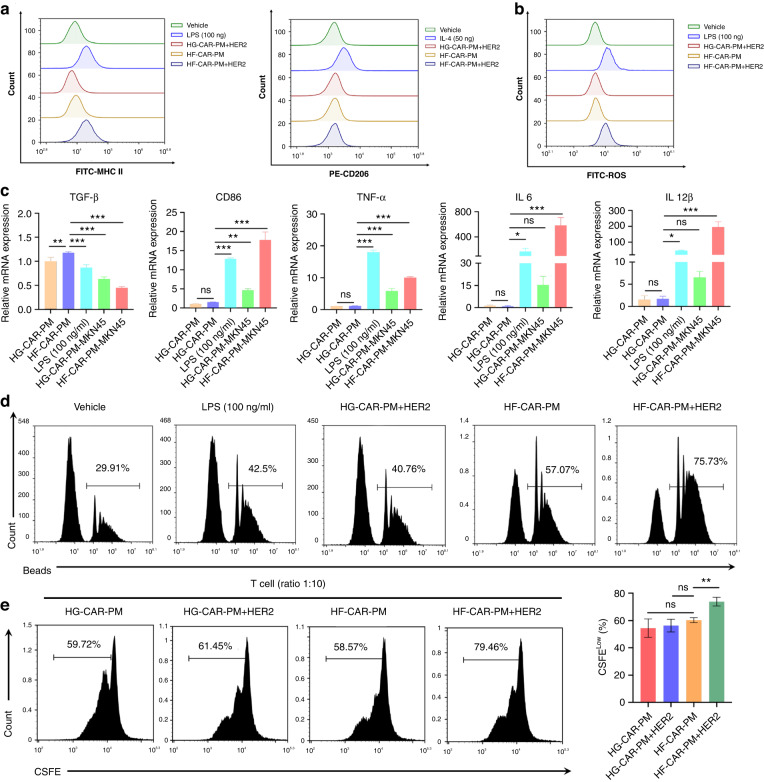
Phenotypic and functional characteristics of CAR-modified PMs under antigen stimulation. **a** Phenotypic markers were detected by treatment with LPS (100 ng/mL), IL-4 (20 ng/mL), HER2 antigen for 48 h. MHC-II to characterise M1polarization and CD206 for M2 polarisation, respectively. **b** Assessment of ROS release from various groups of macrophages using the fluorescent probe DCFH-DA. **c** Expression of genes related to M1 and M2 phenotype was detected by q-PCR after LPS (100 ng/mL) treatment or co-culture with HER2^+^ gastric cancer cells MKN45 for 48 h. **d** The effect of LPS (100 ng/mL), IL-4 (20 ng/mL), and HER2 antigen treatment on phagocytosis of peritoneal macrophages after 48 h. **e** Flow cytometry analysis of T-cell proliferation. HF-CAR-PMs and HG-CAR-PMs were co-cultured with MKN45 for 48 h. Then the cells were co-cultured with CFSE-labelled T cells for 3 days after density gradient centrifugation.

The functional states of macrophages are plasticity and heterogeneity. When recruited into tumours, macrophages shift their functional phenotypes and polarised towards a M2-like tumour-associated macrophages in response to various signals generated from tumour and stromal cells, resulting in acceleration of tumour progression [46, 47]. It is very important for CAR-based macrophages to maintain anti-tumoral phenotype after incubation with tumour cells, in order to keep durable anti-tumoral activity [40]. In this regard, we investigated phenotype characterisation of HF-CAR-PMs after antigen stimulation. As shown in Fig. 2a, stimulated HF-CAR-PMs revealed a tendency toward polarisation by HER2 protein with increased expression of MHC-II and unchanged CD206 expression. Co-culture of HF-CAR-PMs with HER2-positive MKN45 cells resulted in increased expression of CD68, CD86, CCR7 and decreased expression of CD163, inducing a mixed M1/M2 phenotype conversion (Fig. 2c). Activated HF-CAR-PMs can release large amounts of ROS to kill tumour cells and have the greater phagocytic ability (Fig. 2b, d). Moreover, considering macrophages as important antigen-presenting cells, the corresponding antigen-presenting effect was studied in vitro. The capability of antigen presentation by HF-CAR-PMs effectively increased the proliferation of T cells (Fig. 2e). In conclusion, the activation of HF-CAR-PMs tended to promote pro-inflammatory phenotypic transformation, with increased ROS release and significantly upregulated the capacity of phagocytosis and antigen presentation.

### HF-CAR-PMs exhibited antigen-specific cytotoxicity against HER2 + GC cells in vitro

**Fig. 3:**
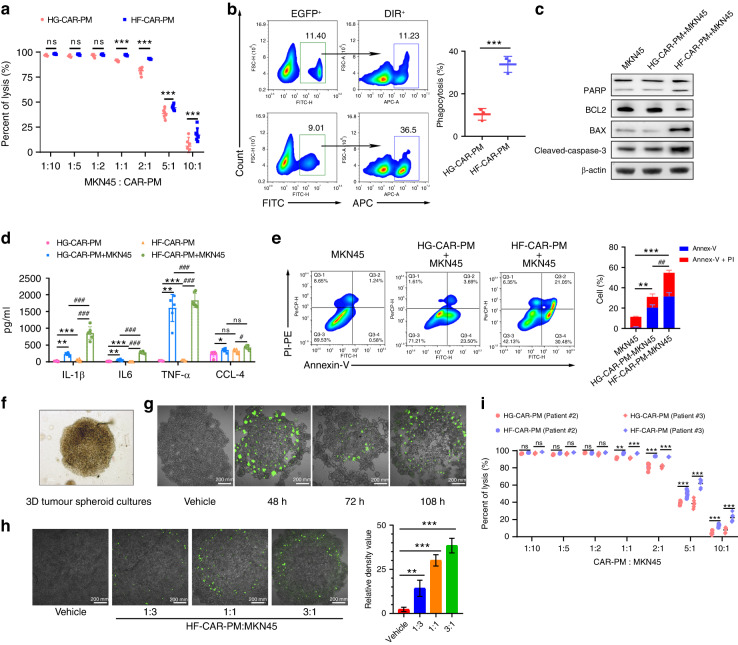
CAR-modified PMs exhibited antigen-specific cytotoxicity against GC cells in vitro. **a** Fluorescence of MKN45 cells at different effector-target ratio (macrophage:tumour = 10:1, 5:1, 2:1, 1:1, 1:2, 1:5, 1:10) was detected by spectrophotometer. Assessment of tumour cell killing using MKN45 cells alone as a baseline. **b** Phagocytosis of GFP-positive macrophages co-cultured with DiR-labelled MKN45 cells. **c** Apoptosis-related protein expression in MKN45 cells co-cultured with HF-CAR-PMs and HG-CAR-PMs. **d** Expression of inflammatory factors after co-culture of MKN45 gastric cancer cells with HF-CAR-PMs and HG-CAR-PMs. **e** Annexin V/PI staining for assessment of MKN45 cell apoptosis in HF-CAR-PMs and HG-CAR-PMs treatment. **f** Microscopic image of MKN45 tumour spheres. **g** Microscopic image of GFP labelled HF-CAR-PMs on MKN45 tumour spheres. **h** Infiltration of GFP labelled HF-CAR-PMs in tumour spheres at different effector/target ratio (macrophage:tumour = 1:3, 1:1, 3:1). The fluorescence intensity was quantified by Image J. **i** Tumor cell killing activity of HF-CAR-PMs and HG-CAR-PMs derived from other two donors. (Statistical analysis was performed by Student’s *t* test when only two value sets were compared. One-way ANOVA followed by Dunnett’s test was used when the data involved three or more groups).

Next, we investigated antigen-specific cellular cytotoxicity of HF-CAR-PMs in vitro. To this end, we first evaluated the expression of HER2 antigen on HGC-27 and MKN45 cells. In addition, breast cancer cell line (MDA-MB-231 and MDA-MB-453) were used as controls (Supplementary Fig. S2A). Our results show that MKN45 cells were HER2-positive, which were selected as target cells for further evaluation. Then, we determined the E/T ratio by cytotoxicity experiments between HF-CAR-PMs and luciferase-labelled HER2-positive/-negative target cells. After 24 h incubation, the cell viability was evaluated by measurement of the level of luminescence, in order to indicate the cytotoxicity, as described before [48]. The results showed that HF-CAR-PMs were of great ability to eliminate HER2-positive MKN45 cells and exhibited in a dose-dependent manner. In addition, the tumour-killing effects were significantly elevated in macrophages after being modified by HF-CAR, rather than by HG-CAR starting from an E/T ratio of 1:10 to 1:1, and the anti-tumoral efficiency was reached to peak at E/T ratio of 1:1 (Fig. 3a). Similar in vitro cytotoxicity was observed in peritoneal CAR macrophages derived from other two donors, Patients #2 and #3 (Fig. 3i). Together, these data indicated that HF-CAR-PMs showed promising anti-tumoral effects against HER2-positive MKN45 cells in vitro.

One key function of macrophage in anti-tumour immunity is presenting tumour antigens to T cells through phagocytosis. We demonstrated that HF-CAR-PMs exhibited greater phagocytosis, the ability of pro-inflammatory factor (TNF-α and IL-1β) release, and the capability to promote apoptosis of MKN45 cells compared to HG-CAR-PMs (Fig. 3b–e). Our study also demonstrated that the activation of HF-CAR-PMs was dependent on the presence of HER2 antigen and the tumoricidal activity was enhanced with higher HER2 expression on the tumour cell surface (Supplementary Fig. S2B–L).

Considering previous reports, we speculated that CAR-based macrophages exhibited good penetration ability in solid tumours. To reveal this speculation, we established 3D tumour spheroid model and assessed the infiltrative effect of HF-CAR-PMs, as described before. Briefly, after the formation of the MKN45 tumour sphere, HF-CAR-PMs were added according to the E/T ratio (1:3, 1:1 and 3:1). We observed that with the increased addition of HF-CAR-PMs, the infiltration proportion within tumour spheres increased (Fig. 3h). At 108 h post addition, the tumour sphere was completely disintegrated at an E/T ratio of 3:1 (Fig. 3g). Collectively, HF-CAR-PMs posed the ability to target HER2-positive tumour cells and inhibited the growth of these tumour cells in vitro.

### HF-CAR-PMs exhibited anti-tumour effects against disseminated MKN45 peritoneal tumours

We constructed a gastric cancer peritoneal carcinomatosis model, for further assessment of targeting and infiltration effect of HF-CAR-PMs in vivo (Supplementary Fig. S3A). MKN45 cells were injected into the peritoneal cavity of nude mice. Two weeks later, groups were treated by DiR-labelled PMs (control group) and HF-CAR-PMs. The region of aggregated HF-CAR-PMs cells and tumour cells overlapped after 48 h of injection, both of which were clustered in the upper and lower abdomen. In contrast, no significant overlap occurred between PMs and tumour cells (Supplementary Fig. S3B). Mice internal organs were explanted for further investigation of macrophages distribution (Supplementary Fig. S3C). DiR-labelled HF-CAR-PMs mainly accumulated in tumour sites compared to other organs, while PMs clustered in the liver, kidney and stomach. Accumulation of PMs was also observed at the tumour site, possibly due to hypoxic recruitment. We next sought to evaluate the persistence of HF-CAR-PMs in mice. The maximum fluorescence signal was detected on day 2 (48 h post-injection) for DiR-labelled HF-CAR-PMs, while the intensity of the signal gradually diminished over time (Supplementary Fig. S3D). In conclusion, these results suggested that HF-CAR-PMs had the potential to penetrate the tumour and did not persist in the peritoneal cavity for a long time.

**Fig. 4:**
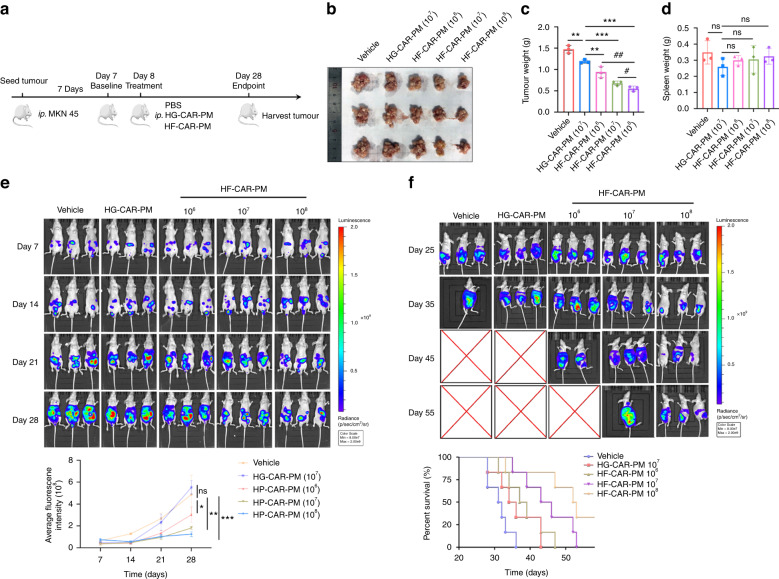
The effect of CAR-modified PMs on tumour growth in vitro. **a** The schematic representation of the experimental design. Male BALB/c nude mice (6–8 weeks old) were injected intraperitoneally with 1 × 10^6^ MKN45-Luc cells/each, and seven days later groups were treated with PBS, 1 × 10^7^ cells/100ul per mouse HG-CAR-PMs cells; low-dose 1 × 10^6^, medium-dose 1 × 10^7^ and high-dose 1 × 10^8^ HF-CAR-PMs cells/100 μL per mouse for the HF-CAR-PMs treatment groups. **b**, **c** Representative image and quantitative analysis of tumour weight in the vehicle, HG-CAR-PMs, and HF-CAR-PMs treatment groups. **d** Quantitative analysis of spleen weight for the vehicle, HG-CAR-PMs, and HF-CAR-PMs treatment groups. **e** Quantitative analysis of fluorescence intensity captured every 7 days for each group. Tumour-bearing mice were injected intraperitoneally with 2 mg of luciferase substrate solution each (Fluorescence intensity range 8 × 10^7^–2 × 10^9^). **f** Representative bioluminescence imaging and survival curve of each treatment group. Statistical analysis by one-way ANOVA with Tukey’s multiple comparison test. *P < 0.05, **P < 0.01 and ***P < 0.001.

Peritoneal carcinomatosis was modelled for further evaluation the antitumor effect of HF-CAR-PMs in vivo (Fig. 4a). To this end, we generated HF-CAR-PMs from Patient #2. Seven days after tumour injection, groups were treated with PBS (vehicle), HG-CAR-PMs cells, HF-CAR-PMs cells (1 × 10^6^, low dose), HF-CAR-PMs cells (1 × 10^7^, medium dose), and HF-CAR-PMs cells (1 × 10^8^, high dose). The results of the in vivo imaging system (IVIS) showed that HF-CAR-PMs inhibited tumour growth (Fig. 4e). Twenty-eight days after treatment, larger numbers of tumour nodules were found in the vehicle and HG-CAR-PMs mice compared with HF-CAR-PMs treatment groups (Fig. 4b). HF-CAR-PMs effectively inhibited tumour growth and the inhibitory effect was dose-dependent (Fig. 4c). It has been indicated that inflammatory response to the tumour progression leads to splenomegaly. There was no statistically significant difference in the spleen weight between the groups of mice (Fig. 4d). As expected, Chimeric antigen receptors promote the infiltration of PMs in peritoneal dissemination model and the in vivo infiltration effect of HF-CAR-PMs was dose-dependent (Supplementary Fig. S3E–G). The impact of HF-CAR-PMs treatment on the survival of mice was also concluded in this study. HF-CAR-PMs decreased tumour burden and prolonged the overall survival of tumour-bearing mice (Fig. 4f). Next, we analysed the phenotypic conversion of HF-CAR-PMs in solid tumours. High expression of TNF-α, IL-1β, NOS2 and CD86 implied the polarisation of HF-CAR-PMs inside HER2+ tumours toward M1 phenotype (Supplementary Fig. S4A, B). By contrast, a mixed M1/M2 phenotype was observed in HG-CAR-PMs treatment group. The mixed phenotype may be caused by the conversion of macrophages to TAM in tumour microenvironment. The secretion of pro-inflammatory factors such as TNF-α, IL-1β and IL-6 regulated the tumour microenvironment and exerted anti-tumour effects (Supplementary Fig. S4C). The elevated MDA suggested the great killing effect of HF-CAR-PMs through the release of ROS (Supplementary Fig. S4D). In short, M1 polarised HF-CAR-PMs exerted anti-tumour effects by secreting pro-inflammatory factors and ROS to promote apoptosis of tumour cells.

### Exploratory toxicology of HF-CAR-PMs

Numerous studies suggest that CAR-T therapy carries the risk of toxicity such as cytokine release syndrome and neurotoxicity. Therefore, the safety of CAR-based immunotherapy has attracted more attention. To investigate our established CAR-based macrophages toxicology in vivo, we conducted systematic evaluation. Consequently, in all mice, there was no statistical difference of the body weights among groups (Supplementary Fig. S5A). Accumulated HF-CAR-PMs were observed in liver with no effects on biochemical and morphological markers of the liver damage in the treatment group compared with vehicle (Supplementary Fig. S5B, C). Meanwhile, no sign of organ injury was shown by organ histopathology (Supplementary Fig. S5D). In addition, there was no significant difference in various immune cells in the peripheral blood of mice (Supplementary Fig. S5E). These results illustrated the safety and feasibility of HF-CAR-PMs in GC treatments.

### HF-CAR-PMs enhanced the therapeutic efficacy of oxaliplatin on HER2 + GC

Increasing clinical investigation indicated that conventional chemotherapeutics promote anti-tumoral response by increasing the immunogenicity of tumour cells and enhancing immune cells infiltration into tumour microenvironment [49–51]. On this basis, we assumed that chemotherapeutics could synergistically enhanced HF-CAR-PMs therapeutic effects. To test this assumption, we established PC model, and generated HF-CAR-PMs from Patient #3 for further evaluation whether HF-CAR-PMs enhanced the anti-tumoral efficacy of oxaliplatin, the first-line chemotherapy agent in GC treatment, as single treatment or combined with HF-CAR-PMs. Firstly, we estimated the inhibitory effects of oxaliplatin on GC cells and PMs, in order to verify whether oxaliplatin suppresses PMs viability. As a result, the IC50 of oxaliplatin against MKN45 cells was 6.8745 μM, while the IC50 of oxaliplatin against PMs was 13.624 μM (Supplementary Fig. S6A, B). In addition, apoptosis detection assay revealed that 5 μM of oxaliplatin did not induce apoptosis in macrophages, providing an optimal oxaliplatin concentration of 5 μM for cells in vitro (Supplementary Fig. S6G). Interestingly, we observed that oxaliplatin treatment in low dosage exhibited a trend for the conversion of macrophages toward M1 phenotype polarisation (Supplementary Fig. S6C, E), whereas had little effects on phagocytic ability and ROS releasing (Supplementary Fig. S6D, F). Altogether, oxaliplatin administered at 5 μM had a certain killing effect on MKN45 cells, and could promote the conversion of M1 phenotype, demonstrating the potential that oxaliplatin synergistically enhanced the anti-tumoral efficacy of HF-CAR-PMs against HER2-positive GC.

The killing and anti-invasive activity of HF-CAR-PMs against HER2-positive MKN45 cells has been demonstrated in our previous study. In order to explore the therapeutic effect of combination with chemotherapy drugs, we further evaluated the in vitro antitumor effect of HF-CAR-PMs combined with oxaliplatin. In vivo experiments were performed using 6–8 weeks old male BALB/c nude mice via injection intraperitoneally with 1 × 10^6^ MKN45 cells. When tumours were palpable, HF-CAR-PMs were injected intraperitoneally once, and oxaliplatin was injected intravenously every 5 days. In vivo imaging was performed every 7 days, the mice were sacrificed after 21 days, the abdominal cavity was dissected, and tumour nodules were taken for analysis. Our results showed that the combination of HF-CAR-PMs and oxaliplatin significantly promoted apoptosis of tumour cells (Supplementary Fig. S7A). The combination treatment of HF-CAR-PMs and oxaliplatin enhanced the tumour-killing effect (Supplementary Fig. S7B). Furthermore, oxaliplatin treatment enhanced secretion of pro-inflammatory cytokine IL-6 and IFN-γ, synergistically promoting HF-CAR-PMs-induced tumour cells killing (Supplementary Fig. S7C). Therefore, the increased anti-tumour activity mainly relied on the promotion of apoptosis and improvement of tumour-killing effect by combination therapy.

**Fig. 5:**
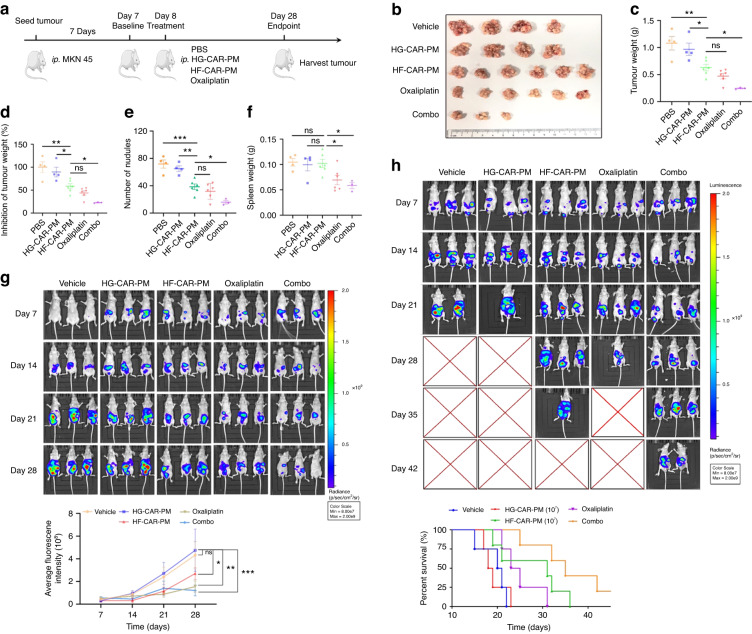
Oxaliplatin combined with HER2-specific CAR macrophages inhibits gastric cancer growth in tumour-bearing mice. **a** Schematic diagram of the intraperitoneal dissemination model and experimental design. BALB/c nude mice were injected with 1 × 10^6^ of MKN45-Luc cells. Seven days later, mice were treated with PBS, HG-CAR-PMs, HF-CAR-PMs, oxaliplatin, and combo therapy (HF-CAR-PMs & oxaliplatin). **b**–**d** Quantification analysis of tumour weight and tumour inhibition rate in each treatment group. **e** Number of tumour nodules in the abdominal cavity of nude mice (nodules <3 mm). **f** Quantification analysis of spleen weight for each treatment group. **g** Tumour growth during treatment. **h** Representative bioluminescence imaging and survival curve of each treatment group.

PC model was constructed to determine the anti-tumour effect of HF-CAR-PMs combined with oxaliplatin in vivo (Fig. 5a). Seven days after tumour injection, groups were treated with PBS (vehicle), HG-CAR-PMs cells (1 × 10^7^), HF-CAR-PMs (1 × 10^7^), oxaliplatin (3 mg/kg), and combination therapy group (1 × 10^7^ of HF-CAR-PMs in combination with 3 mg/kg of oxaliplatin). Oxaliplatin treatment was administered by a single tail vein injection every 5 days, while macrophages were injected on day 7. Twenty-one days post-treatment, larger numbers of tumour nodules were found in the vehicle and HG-CAR-PMs mice compared with other treatment groups (Fig. 5b). Tumours were significantly suppressed in the treatment groups (HF-CAR-PMs, oxaliplatin, and combination therapy group). The tumour growth inhibition rate in the combination treatment group reached 80%, reflecting the synergistic effect of HF-CAR-PMs and oxaliplatin (Fig. 5c–e). No substantial differences were found in the spleen weight between the groups of mice (Fig. 5f). In vivo imaging system (IVIS) showed that HF-CAR-PMs in combination with oxaliplatin possessed the tumour-killing ability and significantly inhibited tumour cell dissemination in the peritoneal cavity (Fig. 5g). In addition, survival data for each group indicated that the HF-CAR-PMs and combination therapy group was found to significantly prolong the survival time of mice (Fig. 5h). Collectively, HF-CAR-PMs derived from different donors showed equally significant anti-tumour activity both in vitro and in vivo, indicating that peritoneal CAR macrophages are of great potential as a potent anti-tumour therapy.

**Fig. 6:**
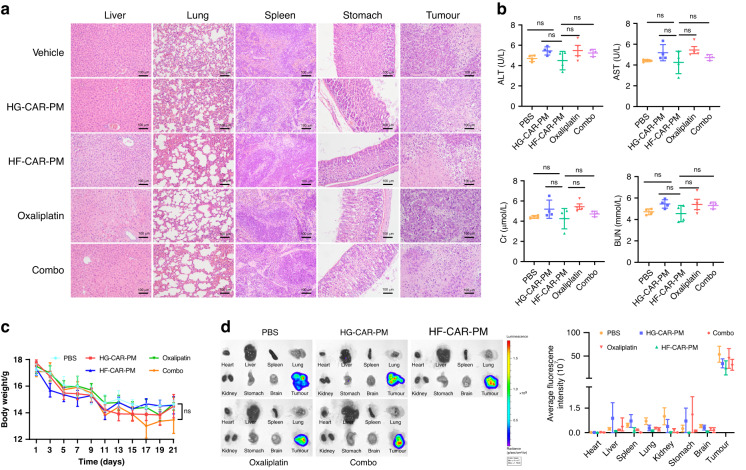
Safety evaluation of CAR-modified PMs in combination with oxaliplatin for solid tumours. **a** Hematoxylin–eosin (H&E) staining of the heart, liver, spleen, lung, kidney, and tumour in each group of mice. **b** Expression of alanine transaminase (ALT), aspartate transaminase (AST), blood urea nitrogen (BUN), and creatinine (Cr) in peripheral blood of mice serum. **c** Body-weight changes in mice receiving different treatment. **d** Representative in vivo images (IVIS) and quantification of luciferase in mice organs.

The safety of the HF-CAR-PMs combined with oxaliplatin treatment was also assessed. The H&E staining images indicated that the HF-CAR-PMs treatment did not cause damage to the liver (Fig. 6a). The Biochemical marker of AST and Cr slightly elevated in the peripheral blood of oxaliplatin-treated mice compared to the vehicle group (Fig. 6b). Moreover, a slight upregulation of BUN and ALT in the HG-CAR-PMs treatment group was also noted. The body weight of mice showed a constant decreasing trend with increasing administration time (Fig. 6c). In all mice, the presence of tumour cells in organs was also quantified by the IVIS system. High expression of fluorescence signal indicated that infiltration and accumulation of HF-CAR-PMs occurred in the liver and the tumours did not metastasise into other organs (Fig. 6d). The above results initially revealed the safety of intraperitoneal injection of HF-CAR-PMs combined with oxaliplatin in the treatment of tumours.

## Discussion

Previous studies have already indicated that tumour invasion and metastasis are the main causes of tumour recurrence [52], which is also a major challenge for radiotherapy [53, 54]. Metastasis to the peritoneal cavity is common for GC patients. The metastasis of GC triggers the formation of malignant ascites fluid and numerous tumour islets distributing to the peritoneum. The whole peritoneal cavity turns into a tumour-promoting microenvironment, accelerating GC metastasis and progression. Emerging evidence suggests that malignant ascites in GC consist of a complex mixture of malignantly transformed cells, immune cells, and stromal cells. As a prominent component in malignant ascites, macrophages exhibit distinct M2-like characteristics, favouring tumour progression [55–57]. In this study, PMs derived from the malignant ascites fluid of GC patients were isolated and characterised as tumour-promoting M2-like phenotype. Given the remarkable immunosuppressive features of M2-like peritoneal macrophage, reciprocal M2 to M1 conversion may be an ideal immunotherapeutic strategy [32, 58]. It has been proven that macrophages with the antitumor state have the potential to combat tumour cells. In addition, antitumor macrophages can also limit tumour growth via the secretion of activating factors and chemokines that stimulate and recruit antitumor cytotoxic T cells [59, 60]. In addition, it is of great significance to fully understand the characteristics of macrophages derived from malignant ascites, and to study their utilisation value.

However, reliable sources and expansion of macrophages applicable to clinical use still remain limited [24, 40]. In this case, malignant ascites-derived macrophages may provide an alternative strategy. Furthermore, it was reported that macrophages are intrinsically more resistant to transduction procedures than T cells and NK cells [18, 61]. In this study, the GFP-only plasmid was transiently transfected into PMs by using Lipo2000 liposomes, and the transfection efficiency was only about 1%. In addition, the lentiviral vector containing GFP gene was purified by a lentivirus packaging system and transfected into PMs. After 3 days of culture, the positive rate of EGFP by flow analysis was about 2%. To overcome the inherent resistance of PMs to lentiviral transfection, we applied Vitamin D3 and NATE™ for the first time to activate macrophages. After activation, it is noteworthy that the transfection efficiency of PMs improved to 30%. Intriguingly, activated CAR-modified PMs by simulating with HER2 overexpressed tumour cells exhibit polarisation toward an anti-tumoral and pro-inflammatory phenotype, meanwhile, promote the proliferation of T cells.

Currently, peripheral intravenous infusion is the main drug delivery strategy for adoptive cell therapy. Injected exogenous macrophages tend to remain in the liver, which may affect the effectiveness of the treatment [40]. Thus, intraperitoneal administration is considered in this study to address those problems. Our study revealed significant antitumor efficacy and safety of intraperitoneal injection of CAR-modified PMs in HER2^+^ gastric cancer mouse peritoneal carcinomatosis models. Recently, autologous macrophages have been applied in many clinical cases for the treatment of solid tumours. These clinical data demonstrated the feasibility and safety of injecting autologous monocyte-derived macrophages, but failed to indicate effective anti-tumour effects [62]. Our preclinical in vivo studies have achieved ideal therapeutic effects in the animal experiment, however, the actual tumour microenvironment in human is more complex. Based on that, the therapeutic effect of CAR-modified PMs in GC patients with peritoneal carcinomatosis needs to be further verified. Thus, in order to verify the curative potential of HF-CAR-PM in GC patients with PC, we are trying to apply for Investigator-Initiated Clinical Trial (IIT) for further exploration.

The discovery of immune checkpoint inhibitors such as programmed cell death ligand-1 (PD-L1) or programmed cell death-1 (PD-1) monoclonal antibodies have improved the overall survival of various types of cancers over the past decade. However, only a limited number of GC patients with peritoneal metastatic can benefit from immune checkpoint blockade therapy [63]. Intraperitoneal chemotherapy has been proven effective in the prevention of peritoneal recurrence in GC [64]. Accumulating evidence suggests that chemotherapy drugs play a surprising role in immune cell reprogramming of the TME in patients with advanced GC, expanding the possibility of chemotherapy to cure cancer. Chemotherapy can directly reprogramme the macrophage phenotype, modulating TAMs towards antitumor effectors [65]. Chemotherapeutic drugs to reprogramme TAMs into an anti-tumour phenotype is regarded as a very promising tumour treatment strategy. As a platinum-based chemotherapeutic agent, oxaliplatin exhibits great anti-tumour efficacy. Oxaliplatin has the capacity to induce immune response with the presence of damage-associated molecular patterns (DAMPs) without causing gastrointestinal inflammation. Sensory peripheral neuropathy is a common adverse effect seen in cancer patients treated with oxaliplatin. However, intraperitoneal administration of CAR-modified PMs combined with oxaliplatin significantly prolong the survival time of mice in this study.

In summary, our study demonstrates for the first time the therapeutic potential of intraperitoneal injection of CAR-modified PMs, and the experimental results suggest that this may be an innovative strategy to ultimately improve the therapeutic efficacy of ACT. In addition, our results warrant further evaluation of the efficacy of a combination of CAR-modified PMs with oxaliplatin in preclinical settings for the treatment of GC. Overall, this study provided an enthusiastic preliminary result and set the stage for future clinical studies on CAR-modified PMs in GC immunotherapy.

## Supplementary information

Supplementary Figures

Supplementary Table 1. mAbs information

Supplementary Table 2. Primers

## Data Availability

All data associated with this study are present in the paper or the Supplementary Materials. Reagents developed in this study will be made available to the scientific community through a material transfer agreement by contacting YB.
