# Supplementary material for: Efficacy evaluation of chimeric antigen receptor-modified human peritoneal macrophages in the treatment of gastric cancer
Source: Br J Cancer. 2023 Jun 29;129(3):551–62. doi: 10.1038/s41416-023-02319-6 (PMC10403530; doi:10.1038/s41416-023-02319-6)
Supplement: Supplementary file 1 — Supplementary Figures [file 41416_2023_2319_MOESM1_ESM.docx]

**Supplementary information**

**Supplementary Figure 1. Validation of the effect of CAR structure activation on macrophage viability**

**
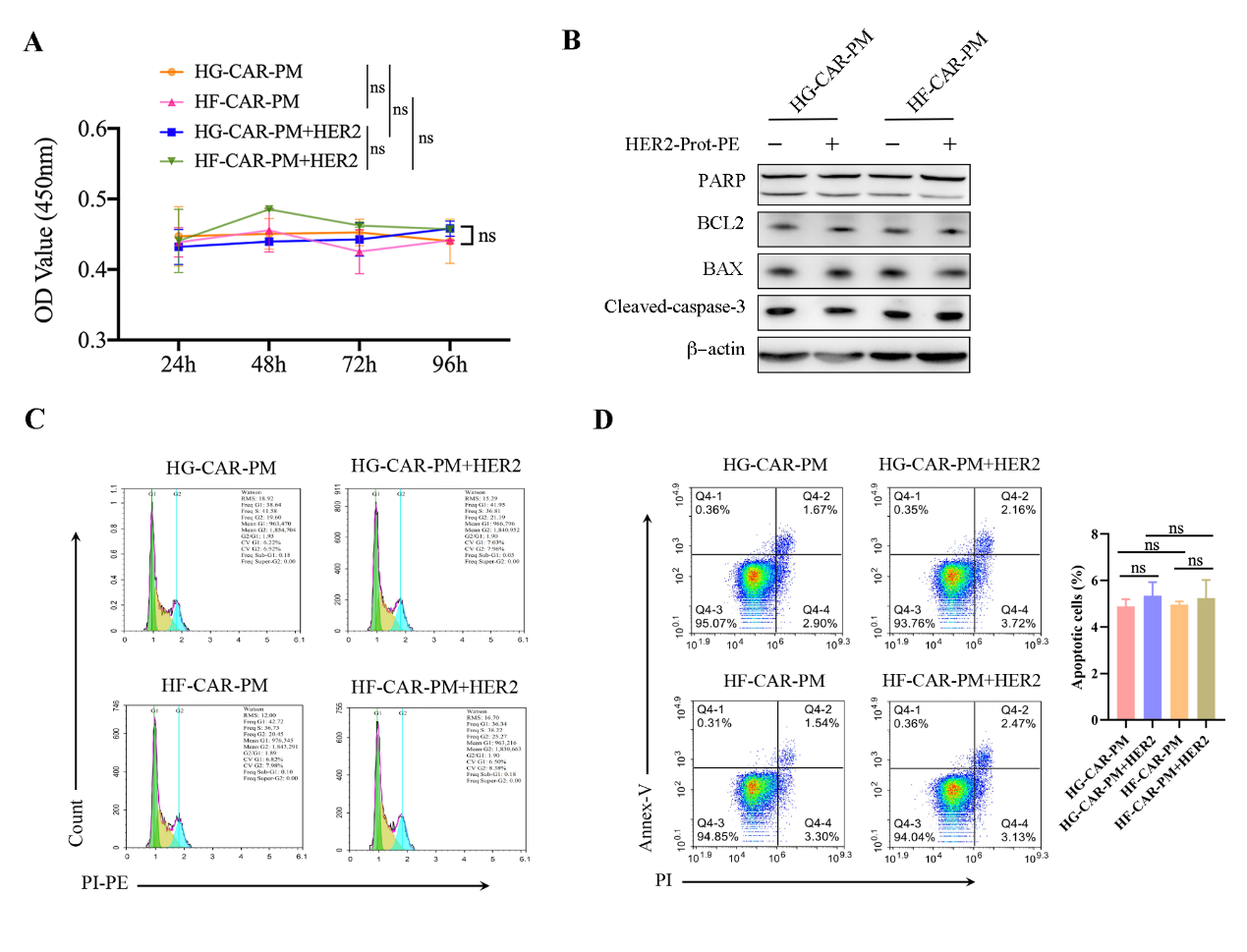
**

**A．**CCK8 assay analysis the cell viability of control PMs and CAR modified PMs before and after HER2 protein activation. **B**. The expression of apoptosis-related proteins in control PMs and HF-CAR-PMs after HER2 protein treatment for 2 days. **C**. Flow cytometry analysis of the cell cycle changes of control PMs and HF-CAR-PMs after HER2 protein stimulation for 2 days. **D**. Flow cytometry analysis of the early and late apoptosis of control PMs and CAR modified PMs after HER2 protein treatment for 2 days.

**Supplementary Figure 2.** **Phagocytic and cytotoxic effects of HF-CAR-PMs on different tumor cells**

**
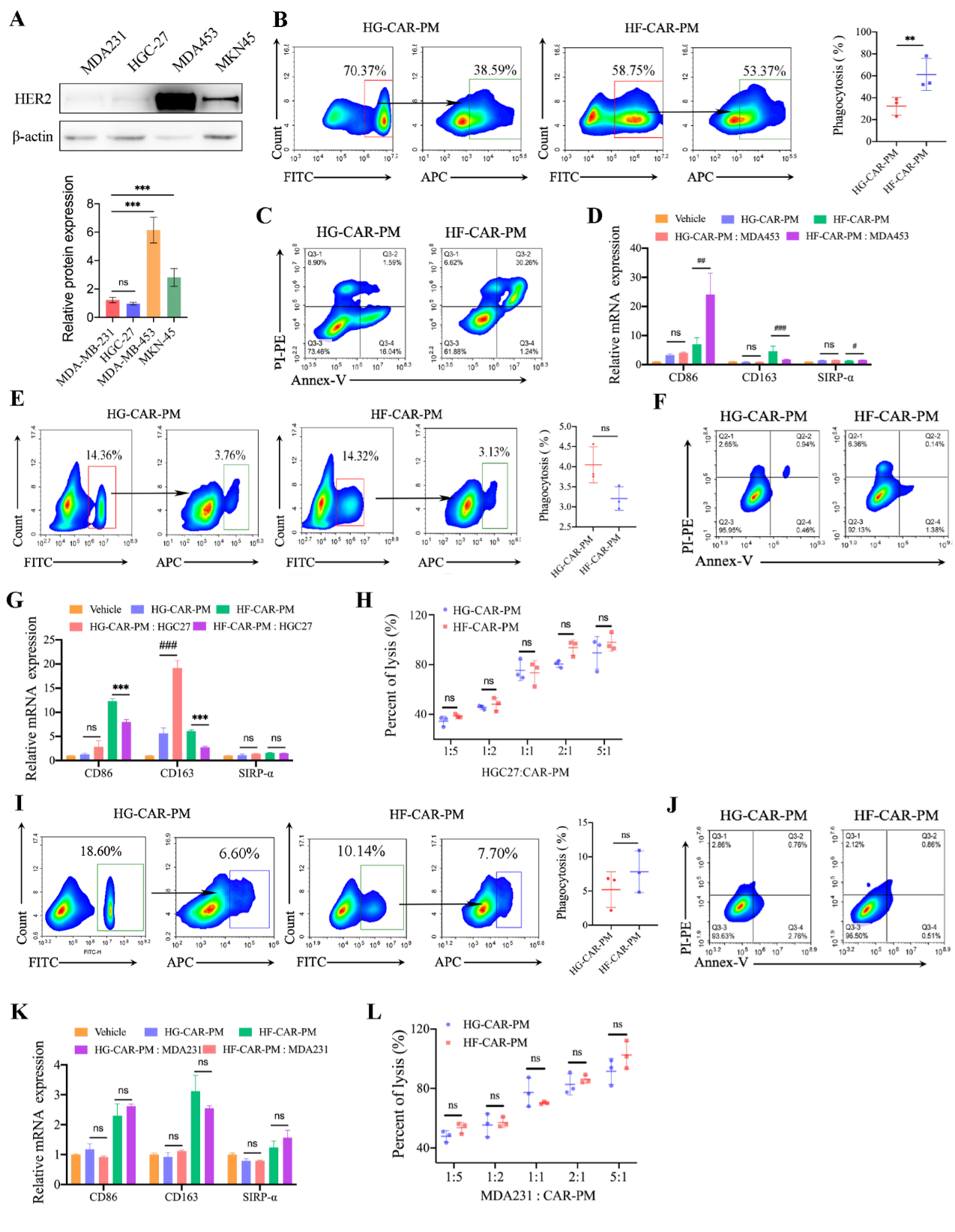
**

**A**. Expression of HER2 on MDA-MB-231, HGC-27, MDA-MB-453, and MKN45 cells. **B**. Phagocytosis of MDA-MB-453 by HF-CAR-PMs. **C**. Apoptotic effect of HF-CAR-PMs on MDA-MB-453 cells. **D**. Effect of MDA-MB-453 cells on phenotypic markers of HF-CAR-PMs. **E**. Phagocytosis of HGC27 by HF-CAR-PMs. **F**. Apoptotic effect of HF-CAR-PMs on HGC-27 cells. **G**. Effect of HGC-27 cells on phenotypic markers of HF-CAR-PMs. **H**. Killing effect of HGC-27 by HF-CAR-PMs (Macrophage: Tumor = 1:5, 1:2, 1:1, 2:1, 5:1). **I**. Phagocytosis of MDA-MB-231 by HF-CAR-PMs. **J**. Apoptotic effect of HF-CAR-PMs on MDA-MB-231 cells. **K**. Effect of MDA-MB-231 cells on phenotypic markers of HF-CAR-PMs. **L**. Killing effect of MDA-MB-231 by HF-CAR-PMs (Macrophage: Tumor = 1:5, 1:2, 1:1, 2:1, 5:1).

**
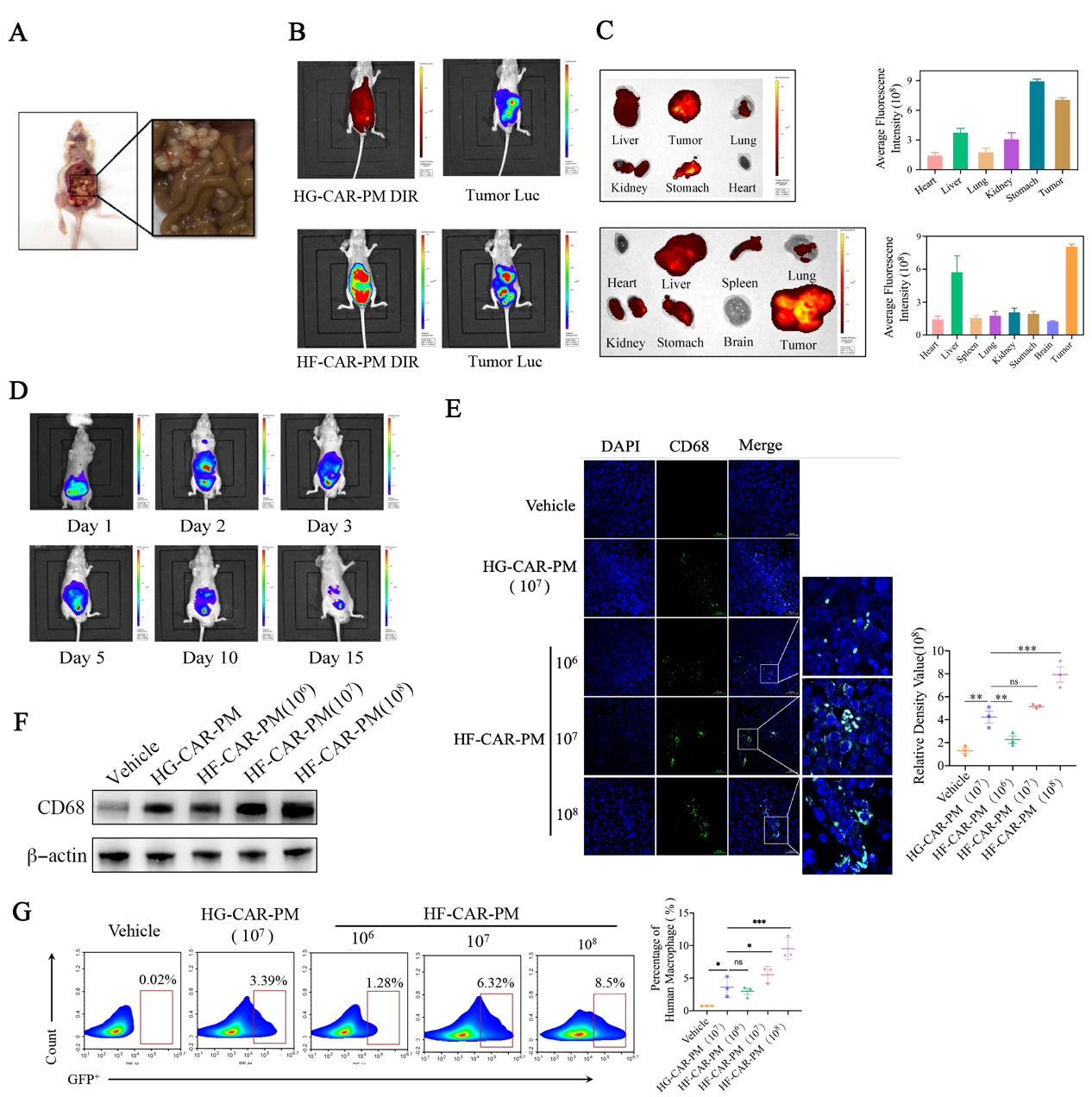
Supplementary Figure 3. Evaluation of targeting, infiltration and persistence of HF-CAR-PMs *in vivo***

**A**. Representative figure of tumor formation in peritoneal cavity of mice 21 days post modeling. **B**. Bioluminescence imaging (BLI) of DiR-labelled HF-CAR-PMs and luciferase-expressing tumor cells for HG-CAR-PMs and HG-CAR-PMs group (48h post-injection). **C**. BLI and quantification of fluorescence intensity for HF-CAR-PMs and tumor cells in different organs. **D**. BLI and quantification of macrophages on Day1, 2, 3, 5,10 and 15 post injection. **E**. Immunofluorescence staining assays analysis the infiltration of PMs in the tumor. **F**. The expression of membrane protein CD68 analysis in the tumor tissue of each group, Image J was used to calculate the gray value. **G**. Flow cytometry analysis of the proportion of HF-CAR-PMs in the tumor suspension after seven days of intraperitoneal injection, and the percentages were calculated.

**
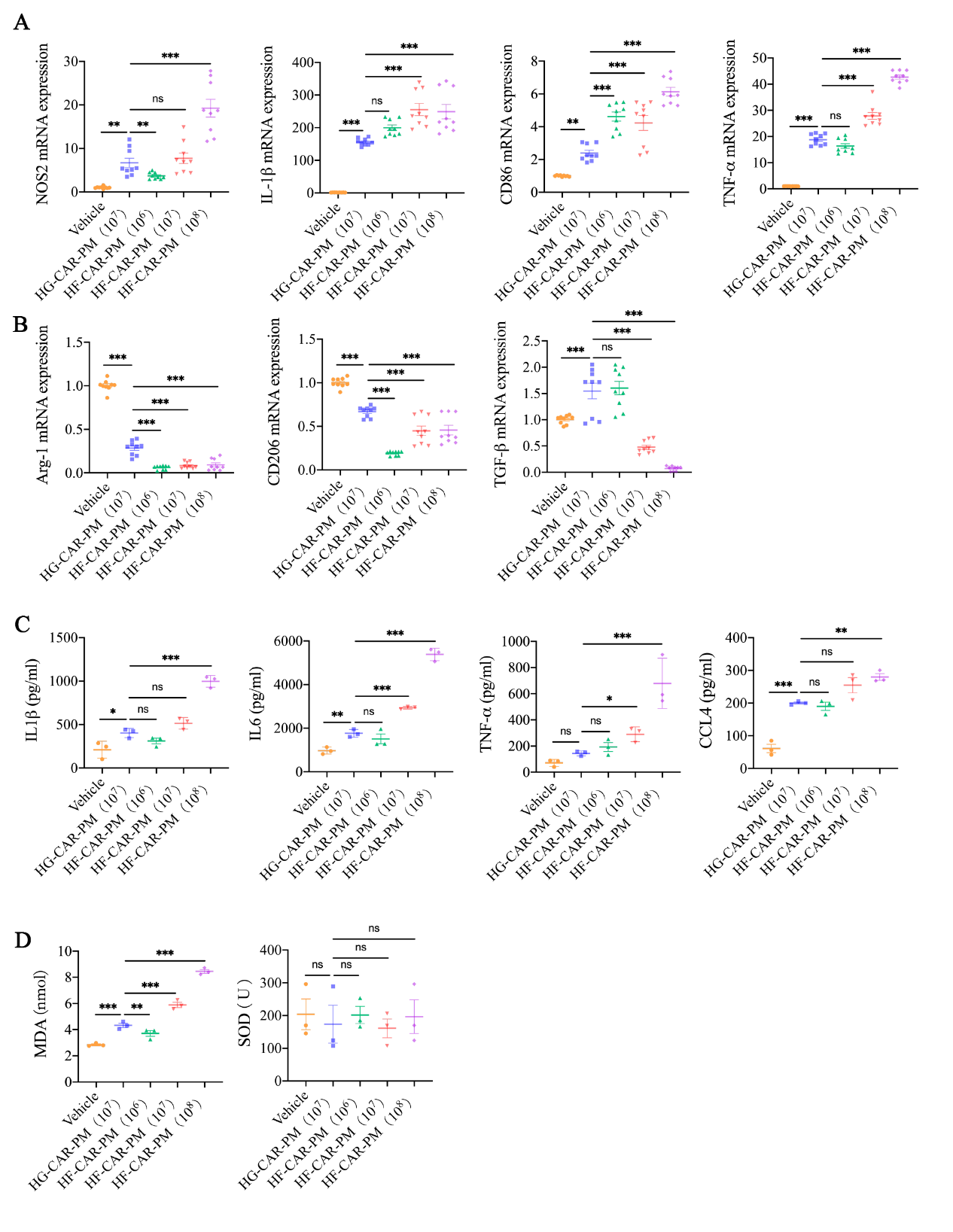
Supplementary Figure 4. Functional and phenotypic analysis of HF-CAR-PMs in solid tumor**

**A**-**B**. Quantification of M1 (NOS2, IL-1β,TNF-α,CD86) and M2 (CD206, Arg1) phenotypic marker of macrophages in tumor. **C**. Quantification of pro-inflammatory cytokines. **D**. Expression of oxidative stress marker SOD and MDA.

**
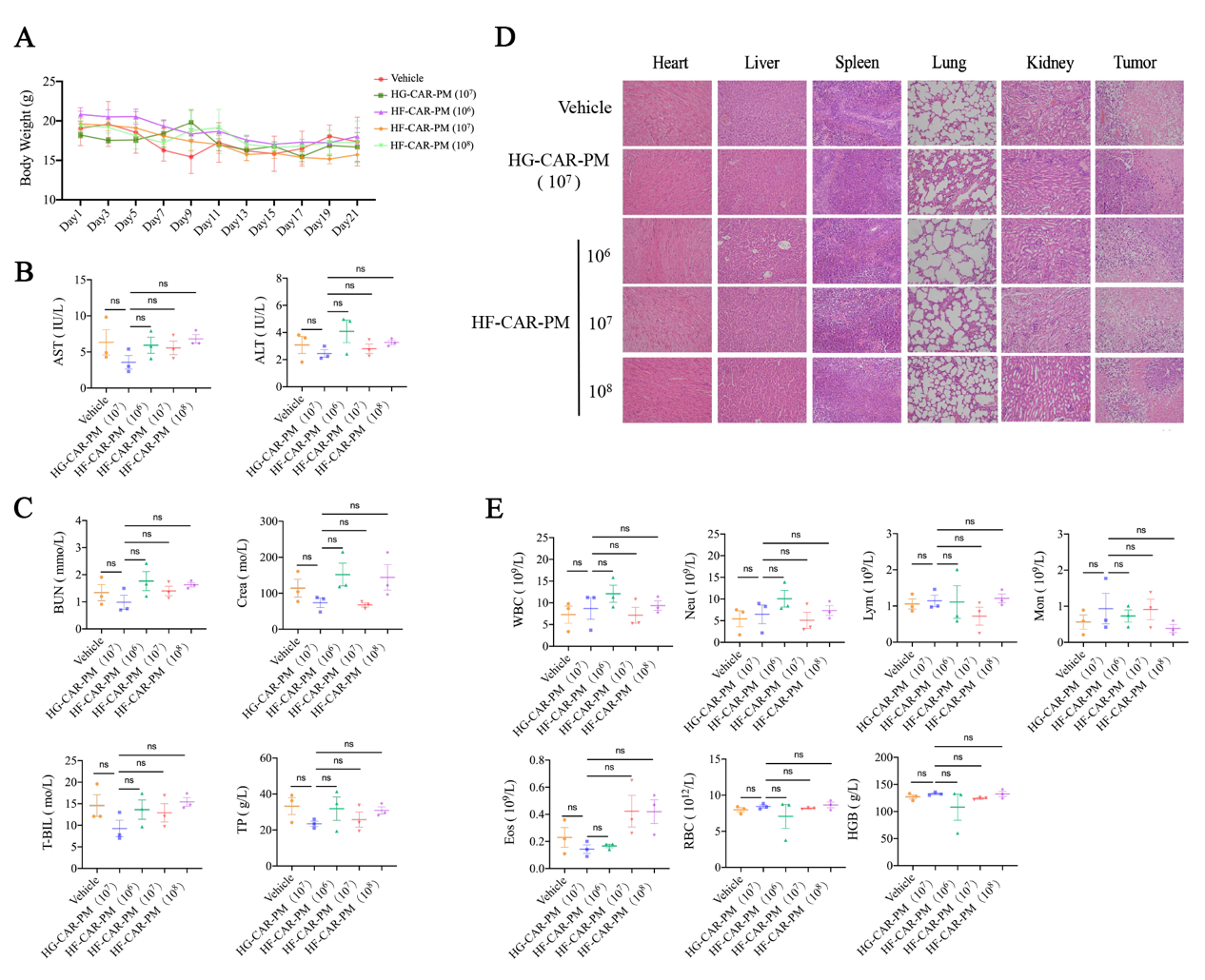
Supplementary Figure 5. Safety evaluation of HF-CAR-PMs therapy in solid tumors**

**A**. Body weight changes of mice receiving different treatment. **B-C**. Expression of ALT, AST, BUN, Cr, T-BIL, TP in peripheral blood of mice. **D**. Representative image of H&E staining for mice organs. **E**. Quantification of WBC, Neu, Lym, Mon, Eos, RBC, HGB.

**Supplementary Figure 6.** **Functional and phenotypic analysis of oxaliplatin on HF-CAR-PMs**

**
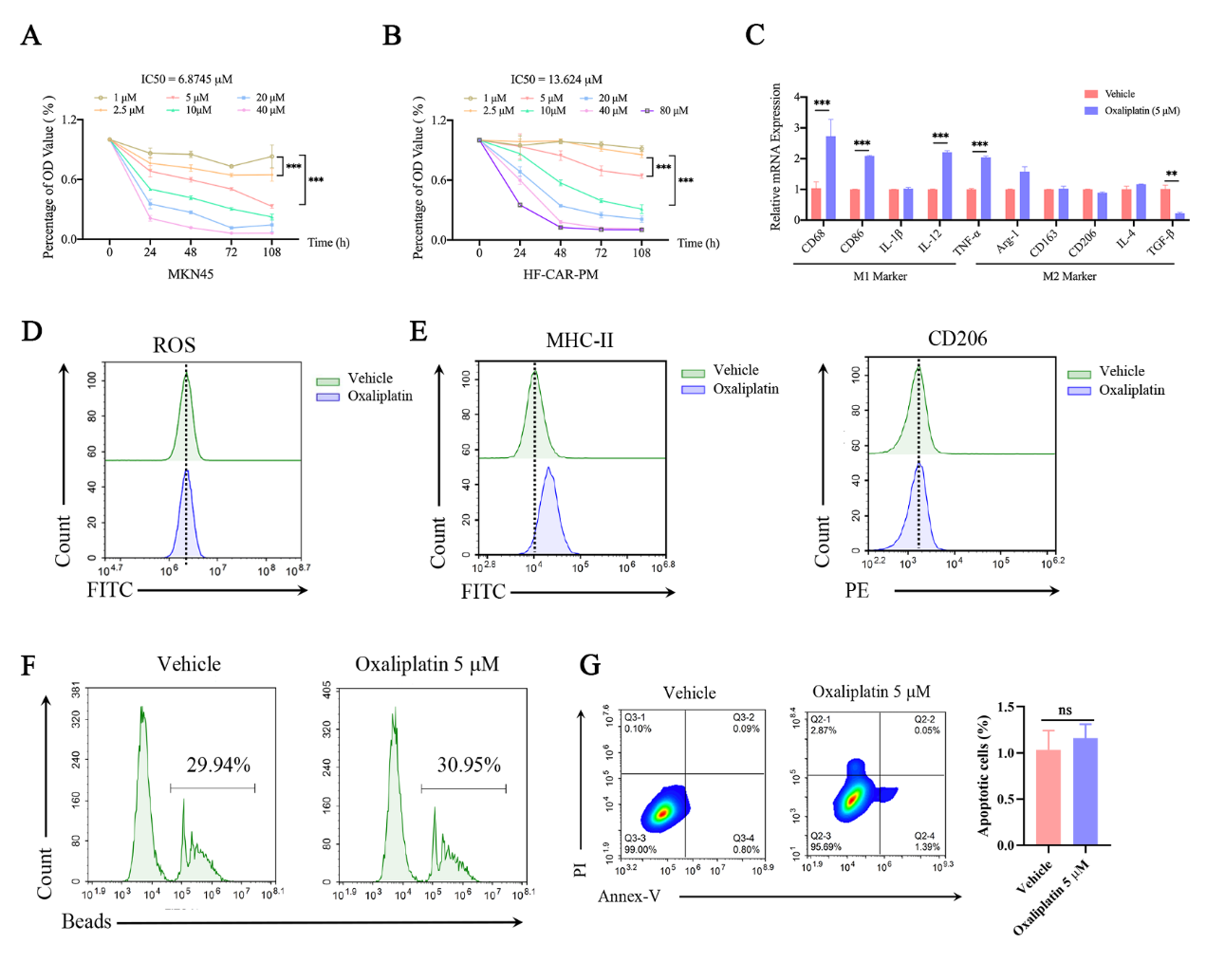
**

**A**. Killing effect of oxaliplatin on MKN45 cells detected by CCK8. **B**. Killing effect of oxaliplatin on HF-CAR-PMs detected by CCK8. **C**. Effect of oxaliplatin on phenotypic markers of macrophages. **D**. Effect of oxaliplatin on ROS release of HF-CAR-PMs. **E**. Determination the effect of oxaliplatin on the phenotype of HF-CAR-PMs phenotype. **F**. Detection of the effect of oxaliplatin on the phagocytic ability of HF-CAR-PMs. **G**. Detection of the effect of oxaliplatin on apoptosis of HF-CAR-PMs.

**
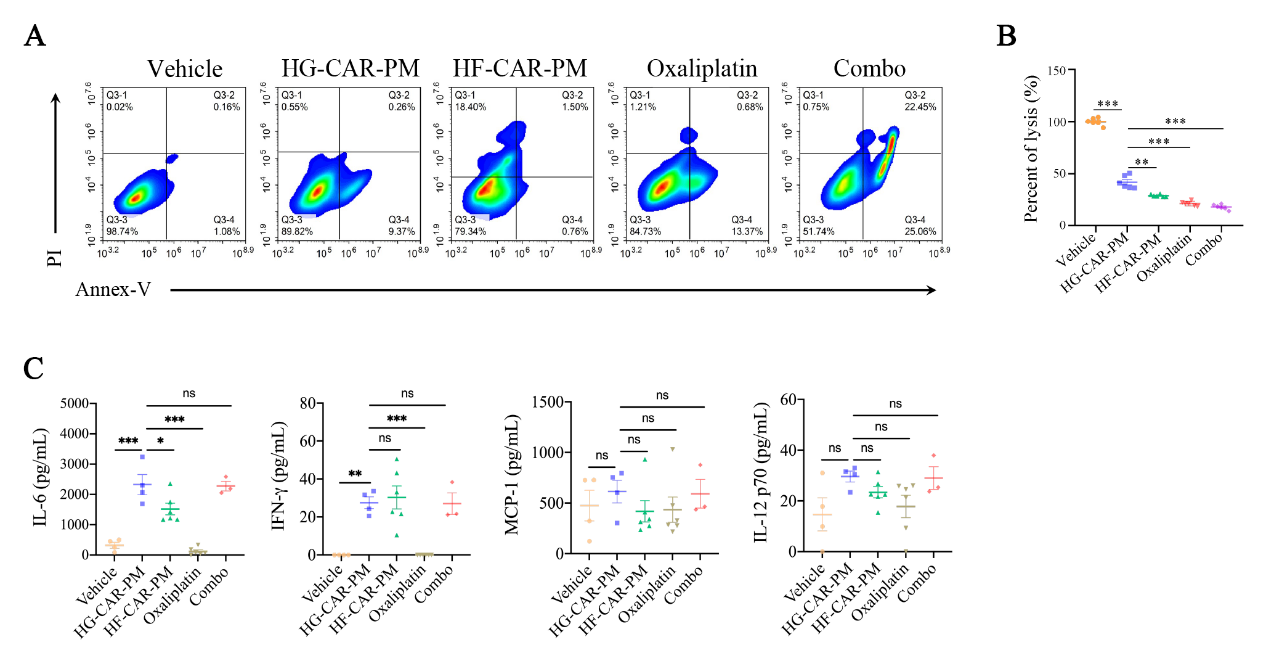
Supplementary Figure 7.** **Killing of HER2-positive cancer cells by HF-CAR-PMs in combination with oxaliplatin**

**A**. Effect of PBS (vehicle), HG-CAR-PMs, HF-CAR-PMs, oxaliplatin and combined therapy on apoptosis of MKN45 cancer cells. **B**. Quantification analysis of tumor killing effect on different treatment group (Macrophages: Tumor = 10:1). **C**. Expression of inflammatory factors after co-culture of MKN45 gastric cancer cells with different treatment.
